# Supplementary material for: Asynchronous magnetic resonance elastography: Shear wave speed reconstruction using noise correlation of incoherent waves
Source: Magn Reson Med. 2022 Oct 27;89(3):990–1001. doi: 10.1002/mrm.29502 (PMC9792433; doi:10.1002/mrm.29502)
Supplement: Supplementary file 1 — DATA S1 MATLAB code used to generate the numerical simulations of Figure 1 [file MRM-89-990-s002.zip › k-Wave/helpfiles/example_tvsp_doppler_effect.html]

The Doppler Effect Example (k-Wave)


# The Doppler Effect Example

This example demonstrates the doppler effect in which a stationary sensor point records a shift in frequency as a moving source travels past. It builds on the Monopole Point Source In A Homogeneous Propagation Medium Example.

- Open the file in the MATLAB Editor
- Run the file in MATLAB

## Contents

- Creating a moving source

## Creating a moving source

The moving source is created by using a line of source points and then gradually moving the active points along this line. To create a smoother source transition from point to point, the source strength is interpolated between pairs of points using linear interpolation. The input pressure signal to each source point along with the total input pressure is plotted below.

A snapshot of the moving source is given below. In this example the source is moving to the right. Notice that the wavefronts are slightly closer together on the right hand side than on the left.

As the source approaches the receiver, the amplitude gradually increases and the perceived frequency is shifted upwards according to the relative velocity between them. The amplitude of the recorded signal reaches a maximum when the source is adjacent to the receiver. The amplitude then gradually decreases as the source moves away, with the perceived frequency similarly shifted downwards. The shift in frequency can clearly be seen by analysing the parts of the time series corresponding to the approach of the source to the receiver and its retreat away again.
